# Co-occurrence of Asthma and the Inflammatory Bowel Diseases: A Systematic Review and Meta-analysis

**DOI:** 10.1038/s41424-018-0054-z

**Published:** 2018-09-24

**Authors:** M. Ellen Kuenzig, Kirles Bishay, Richard Leigh, Gilaad G. Kaplan, Eric I. Benchimol, Crowdscreen SR Review Team

**Affiliations:** 1 Children’s Hospital of Eastern Ontario (CHEO) Inflammatory Bowel Disease Centre, Division of Gastroenterology, Hepatology & Nutrition, Children’s Hospital of Eastern Ontario, Ottawa, Canada; 2 Faculty of Medicine, University of Ottawa, Ottawa, Canada; 3 Department of Medicine, University of Calgary, Calgary, Canada; 4 Department of Community Health Sciences, University of Calgary, Calgary, Canada; 5 Department of Pediatrics and School of Epidemiology and Public Health, University of Ottawa, Ottawa, Canada

## Abstract

**Background:**

Inflammatory bowel diseases (IBD) and asthma share genetic and environmental risk factors. Consequently, several observational studies have explored an association between IBD and asthma. We systematically reviewed and summarized the literature on the co-occurrence of asthma and IBD.

**Methods:**

MEDLINE and EMBASE (to April 2017) were searched to identify observational studies on the association between asthma and IBD. Relative risks (RR) were pooled using random effects models. Heterogeneity was assessed using the *I*^2^ and Cochran *Q* statistics. Meta-regression based on study design, source of patients (population-based vs. tertiary-care center) and study location was conducted to explain between-study heterogeneity.

**Results:**

Eighteen studies were identified (15 Crohn’s disease, 15 ulcerative colitis (UC)). Asthma was associated with both Crohn’s disease (pooled RR 1.30, 95% confidence interval (CI) 1.16–1.47, *I*^2^ = 88%) and UC (RR 1.34, 95% CI 1.24–1.44, *I*^2^ = 93%). The study design and source of patients and study location explained between-study heterogeneity in Crohn’s disease, but not UC.

**Conclusion:**

Asthma is associated with both Crohn’s disease and UC. Additional research is needed to determine if one disease influences the risk of developing the other or if the frequent co-occurrence of these diseases result from shared genetic, environmental, and microbial risk factors.

## Background

Inflammatory bowel disease (IBD) and asthma are both immune-mediated diseases that may be rooted in common pathology, as well as shared genetic and environmental risk factors. Aberrant epithelial barrier function in the lung and gastrointestinal tract, as well as abnormal immune responses to environmental factors and pathogens characterize asthma and IBD^1–7^. The ‘hygiene hypothesis’ has been proposed for both IBD and asthma, and postulates that children growing up in relatively sterile environments are more likely to develop chronic immune-mediated diseases later in life. Moreover, disruption of early life intestinal microbiota may exacerbate risk of disease development. For example, asthma and IBD are more common among individuals exposed to antibiotics early in life, while breastfeeding decreases the risk of both diseases^8–10^. Lack of exposure to enteric pathogens early in life may increase the risk of developing immune-mediated diseases, including both asthma and IBD^11,12^. IBD and asthma also share susceptibility genes, including SMAD3 and IL-23R^13–15^. Both IBD and asthma have become increasingly common in the Western world during the 20th century and these trends are now being echoed in developing countries^16–20^.

A previous systematic review and meta-analysis found that individuals with asthma were more likely to have a co-occurring gastrointestinal or urinary condition, but did not specifically evaluate the association between asthma and IBD^21^. There is growing epidemiologic evidence that asthma and IBD frequently co-occur^22–29^ and a family history of one disease may influence the risk of developing the other^30,31^. However, studies evaluating the association between these two diseases have used heterogeneous study designs. Specifically, studies have differed in terms of (1) the relative timing of the two diagnoses and (2) the age of participants included in the study. These two factors have resulted in differing conclusions about the co-occurrence of asthma and IBD.

We conducted a systematic review and meta-analysis to summarize and quantify the association between IBD and asthma, as well as evaluating the impact of study and patient characteristics on the association between these two diseases.

## Methods

This systematic review is based on a previously registered protocol^32^ and is reported in accordance with the Preferred Reporting Items for Systematic Reviews and Meta-Analyses (PRISMA) guidelines^33^.

### Study identification and selection

We searched MEDLINE, including articles available as online only ahead of print, (1946–1 April 2017) and EMBASE + EMBASE Classic (1947–1 April 2017) for observational epidemiologic studies evaluating the association between asthma and IBD (Crohn’s disease, ulcerative colitis (UC), and IBD-type unclassified (IBD-U)). Search terms for IBD and asthma were grouped using the Boolean operator “AND.” The complete search strategy is outlined in Supplementary Table 1. In addition, we hand-searched: (1) references of included studies and relevant review articles; (2) conference abstracts for major gastroenterology meetings (Digestive Diseases Week, American College of Gastroenterology, and United European Gastroenterology Week) and major thoracic meetings (American Thoracic Society Meeting, European Respiratory Society, and American Academy of Chest Physicians Conference) from 2013 to 2017. The review of abstracts and articles identified for full-text review was conducted via crowd sourcing using CrowdScreen SR^34^. Crowdsourcing has previously been shown to increase the efficiency of the systematic review process, while maintaining high accuracy during the review process^34,35^. Prior to screening, each member of the CrowdScreen SR Review team was asked to review a test set of 15 abstracts identified by the principal investigator (MEK). The test set included a mix of abstracts that should be included in full-text review and abstracts that should be excluded from further review. Feedback was provided on the accuracy of their initial reviews to enhance performance. Members of the review team were medical students or undergraduate/graduate students in health sciences and all completed the initial test set with a high degree of accuracy. Each abstract and full-text was reviewed independently by at least two members of the review team. Discrepancies for both the abstract and full-text review stages were resolved by MEK.

Studies eligible for inclusion were observational epidemiologic studies (case-control, cohort, or cross-sectional studies) that either compared the rate of asthma in patients with and without IBD or the rate of IBD in patients with and without asthma. No restrictions were placed on date of publication, language, or region of study. Studies reporting on any subtype of IBD (Crohn’s disease, UC, or IBD-U) were included. Case reports and case series were excluded, as were studies reporting on the association between pulmonary function tests or other respiratory illnesses with IBD. If multiple studies reported on the same cohort of patients, the study with the most complete cohort of patients was selected for inclusion in the study.

### Data extraction and risk of bias

The following information was extracted from included studies independently by two investigators (MEK, KB) using a piloted data extraction form in REDCap electronic data capture tools hosted at the Children’s Hospital of Eastern Ontario:^36^ study characteristics, including the country and years in which the study was conducted; study design; method of identifying and recruiting individuals to participate in the study; the association between asthma and IBD (crude and adjusted, where possible, and the confounders adjusted for in the model); definitions used to identify and/or confirm cases of asthma and IBD; the age of study participants; the timing of exposure relative to the study outcome; and characteristics of individuals included in each study. Discrepancies were resolved by EIB. The risk of bias in individual studies was determined using the Newcastle-Ottawa Scale^37^.

### Study design and outcomes

The primary outcomes of our meta-analysis were the association between asthma and either Crohn’s disease, UC, or IBD-U. All analyses were conducted separately for Crohn’s disease, UC, and IBD-U. For the primary analysis, no restriction was placed on the timing of one diagnosis relative to the other.

Sensitivity analyses evaluating the temporal associations between asthma and IBD were conducted. Specifically, we conducted two sensitivity analyses in which included studies were limited to (1) those in which the diagnosis of asthma preceded the diagnosis with IBD; and (2) those in which the diagnosis of IBD preceded the diagnosis with asthma. Subgroup analyses were conducted based on the age of diagnosis of IBD, defined according to the Montreal classification (pediatric-onset: ≤16, young adult-onset: 17–40, and older adult-onset: >40)^38^ and/or asthma.

### Statistical analysis

Analyses were conducted separately for the association between asthma and (1) Crohn’s disease; (2) UC; and (3) IBD-U. Relative risks (RR) and their 95% confidence intervals (CI) were pooled to estimate the association between asthma and IBD. Random effects models were used to account for expected heterogeneity across study designs. The most adjusted estimate was used. Odds ratios were assumed to approximate the RR due to the rare prevalence of IBD and asthma. We performed a sensitivity analysis separating case-control from cohort studies.

Between-study heterogeneity was assessed using the *I*^2^ statistic and the Cochran *Q* statistic with *p* < 0.1 being considered statistically significant. Meta-regression was conducted to explore sources of study heterogeneity based on the source of patients involved in the study (population-based vs. recruited from tertiary-care center) and the country in which the study was conducted.

All statistical analyses were conducted using the meta and metafor packages in R Software version 3.4.2^39–41^.

## Results

### Description of included studies

**Fig. 1:**
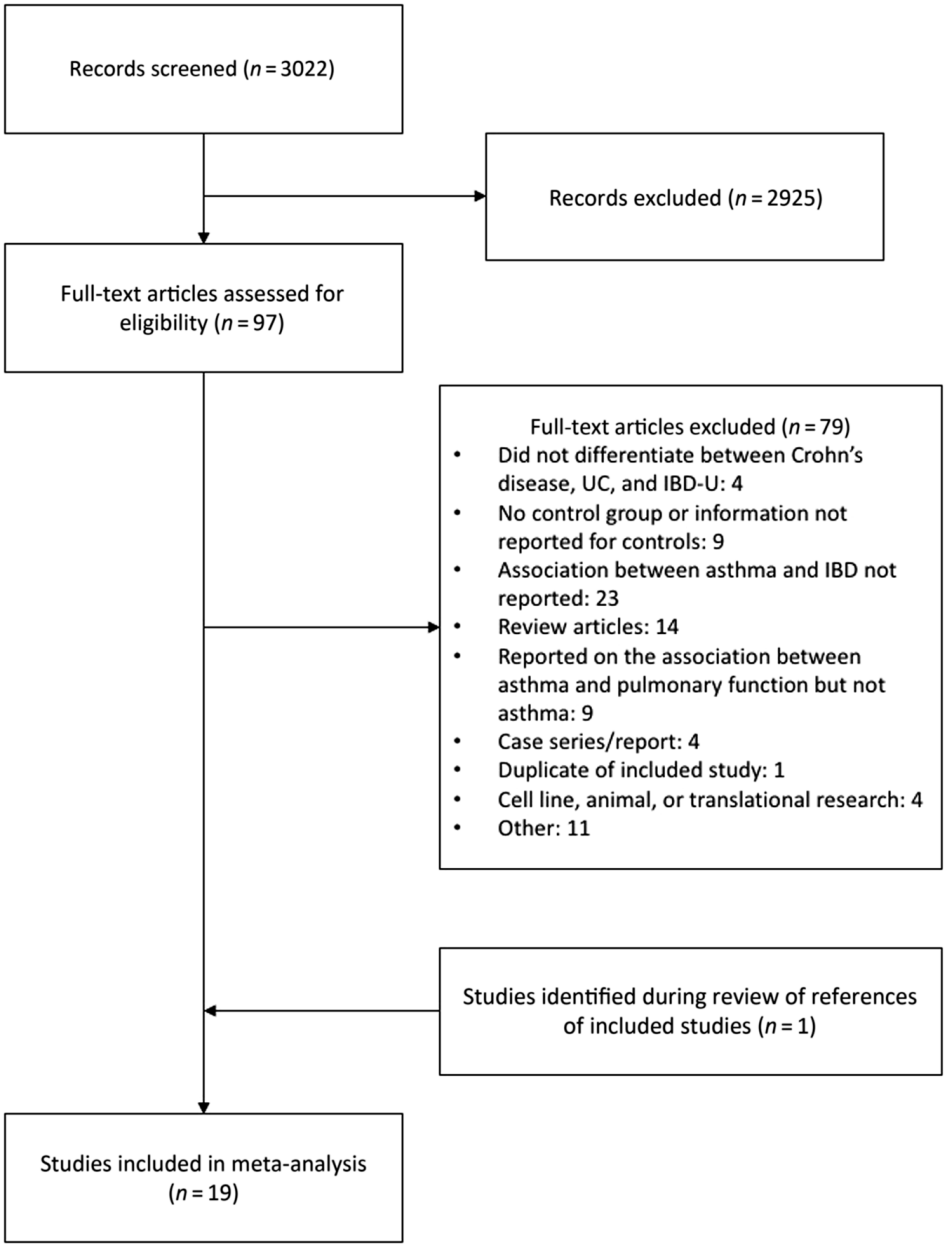
PRISMA flow diagram .

There were 3975 citations identified from the search of MEDLINE and EMBASE. After removing duplicates, 3022 references remained. Of the 97 studies identified for full-text review, 18 of these were included in the meta-analysis (Fig. 1). One additional study was identified after reviewing references of included studies. Fifteen studies reported the association between asthma and Crohn’s disease^22–24,26–29,42–49^. Sixteen studies reported the association between asthma and UC^22–24,26–30,42–44,48–52^. One study reported on the association between asthma and IBD-U^27^. Characteristics of included studies are described in Table 1.

**Table 1:** Characteristics of included studies

Study	Country	Study design	Data source (years of study)	Definition of IBD	Definition of asthma	Relative timing of diagnoses	Type of IBD	Matched variables and covariates	Age of participants	Sample size
Bernstein^23^	Canada	Matched case-control	Provincial health administrative data (1984–2003)	Validated algorithm	≥5 health care contacts for asthma	Asthma diagnosed before or after IBD	CD, UC	Matched on age, sex, and rural/urban residence	Pediatric and adult	IBD: 8072 Controls: 80,489
Boneberger^52^	Chile	Case-control	Tertiary-care center (2009–2010)	Routine clinical practice	Not specified	Not specified	UC	Adjusted for age and sex	Range: 6 to 45 years at study entry	IBD: 52 Controls: 174
Brassard^42^	Canada	Retrospective cohort	Provincial health administrative data (2001–2006)	Externally validated algorithm	≥3 prescriptions for respiratory medication within 1 year, on at least two separate occasions; the third prescription must have occurred at ≤40 years of age	Asthma diagnosed before IBD	CD, UC	Incidence rates were standardized based on age and sex	≤40 years at asthma diagnosis	Asthma: 136,178^a^
D’Arienzo^50^	Italy	Case-control	Tertiary-care center (1998)	Standard diagnostic criteria	Standard diagnostic criteria	Not specified	UC	None	Range: 16 to 69 years	IBD: 50 Controls: 50
D’Arienzo^51^	Italy	Matched case-control	Tertiary-care center (2000)	Standard diagnostic criteria	Standard diagnostic criteria	Not specified	UC	Matched controls were partners of cases	Adult	IBD: 45 Controls: 37
Gearry^24^	New Zealand	Frequency matched case-control	Canterbury IBD Study (2003–2005)	Standard diagnostic criteria	Not specified	Not specified	CD, UC	Frequency matched based on age (at recruitment) and sex. Adjusts for family history of IBD, smoking status, age, social class at birth, sex, and ethnicity	Adult	IBD: 1291 Controls: 600
Hammer^43^	United Kingdom	Matched case-control	Tertiary-care center (1952–1965)	Standard diagnostic criteria	Questionnaire or interview	Not specified	Colonic CD, UC	None	Not specified	IBD: 243 Controls: 319
Hemminki^28^	Sweden	Prospective cohort	National health administrative data (1964–2007)	Hospitalization with an ICD code for IBD	Hospitalization with an ICD code for asthma	IBD diagnosed after asthma	CD, UC	Standardized incidence rates used expected number of cases based on age, sex, year, region, and socioeconomic status	Children and adults	Asthma: 148,295^c^
Kappelman^44^	United States	Matched case-control	PharMetrics Patient-Centric Database (2003–2004)	Validated algorithm	ICD code for asthma	Not specified	CD, UC	Matched on age, sex, health plan type, and geographic region	Children only	IBD: 1242 Controls: 3353
Kuenzig^22^	Canada	Case-control	Population-based health administrative data (1994–2010)	Validated algorithm	Externally validated algorithm	1. Asthma before IBD 2. Asthma diagnosed before or after IBD	CD, UC	Age, sex, rural/urban residence, socioeconomic status	Children and adults	IBD: 5464 Controls: 402,800
Livnat^45^	Israel	Case-control	Tertiary-care center (2008–2009^b^)	Standard diagnostic criteria	Self-report	Not specified	CD	None	Children and young adults	IBD: 23 Controls: 24
Myrelid^46^	Sweden	Matched case-control	Cases: Unclear controls: Southeastern Region Population Registry (2000)	Standard diagnostic criteria	Self-report	Not specified	CD	Age, gender, place of residence, and other atopic manifestations (allergic rhinitis, eczema)	Range: 18 to 50 years	IBD: 275 Controls: 777
Nakamura^30^	Japan	Matched case-control	Cases: Patients receiving financial aid from the Japanese government for the treatment of UC (1988–1990) Controls: Patients on the roster of a health check-up program	Diagnosis of UC by treating physician, and confirmation of diagnosis by independent group for approval of financial aid	Self-report questionnaire	Asthma before IBD	UC	Matched on age and sex	Children and adults	IBD: 384 Controls: 384
Neilly^47^	Scotland	Matched case-control	Not specified	Not specified	Previous physician-diagnosed asthma and/or history of persistent or episodic wheeze with breathlessness, responsive to bronchodilator therapy	Not specified	CD	Matched on age, sex, and smoking history	Adults	IBD: 29 Controls: 29
Peng^29^	Taiwan	Frequency matched retrospective cohort	National health administrative data (2000–2011)	ICD codes for IBD	ICD code for asthma and treated with inhaled corticosteroids, systemic corticosteroids, or inhaled short-acting ß2 agonists	Asthma diagnosed after IBD	CD, UC	Frequency matched on age, sex, and index year. Adjusted for age, sex, and other comorbidities (rhinitis, chronic sinusitis, atopic dermatitis, and chronic obstructive pulmonary disease)	Adults	IBD: 319 Controls: 807
Pugh^48^	UK	Matched case-control	Tertiary care center; Ileostomy Association	Standard diagnostic criteria	Self-report	Not specified	CD, UC	Some controls were partners of cases. Others were matched to cases based on age and sex	Not specified	IBD: 500 Controls: 500
Raj^49^	UK	Cohort	Tertiary-care center (1995–2005)	Standard diagnostic criteria	Consistent clinical picture with objective evidence of variable outflow obstruction and/or airway hyper-responsiveness	IBD preceded the onset of respiratory disease in all cases but 1	CD, UC	None	Mean age: • UC: 61 • CD: 60	Asthma: 893^a^
Virta^26^	Finland	Matched case-control	National health administrative data (1994–2010)	Received special reimbursement for IBD	Received special reimbursement for asthma	Asthma diagnosed before IBD	CD, UC	Matched on date of birth, sex, and place of residence	Pediatric	IBD: 595 Controls: 2380
Weng^27^	United States	Matched case-control	Kaiser Permanente Medical Care Program (1996–2005)	≥2 inpatient or outpatient ICD codes for IBD	≥2 inpatient or outpatient ICD codes for asthma	Asthma could be diagnosed either before or after the diagnosis with IBD	CD, UC, IBD-U	Matched on age, sex, and length of enrolment in health maintenance organization. Adjusted for smoking.	Children and adults	IBD: 12,601 Controls: 50,404

*CD* Crohn’s disease, *IBD* inflammatory bowel disease, *IBD-U* inflammatory bowel disease type unclassified, *ICD* International Classification of Diseases, *UC* ulcerative colitis

^a^The risk of IBD in patients with asthma was compared to the previously reported risk of IBD in the general population

^b^Years of study obtained from study protocol on clinicaltrials.gov

^c^The risk of IBD in patients with asthma was compared to the number of cases that would be expected if there was no association between asthma and IBD

### Risk of bias of included studies

The risk of bias among included case-control and cohort studies is summarized in Supplementary Tables 3 and 4, respectively. The majority of studies were conducted using population-based health administrative data or tertiary-care studies enrolling consecutive patients. Bias may have been introduced to case-control studies based on the controls included in the studies: one study included hospital staff^50^, one included partners of cases^51^, four included hospitalized patients or patients visiting a clinic for reasons not related to IBD or asthma^30,43,45,52^, and one included a mix of partners of cases and non-IBD patients^48^. Nine case-control studies did not explicitly report that controls did not have IBD^24,30,43,45–48,52,53^. Two of the four cohort studies included compared the frequency with which asthma and IBD co-occurred to previously published estimates of the rate of either asthma or IBD in the general population^42,49^.

### Association between asthma and IBD

**Fig. 2:**
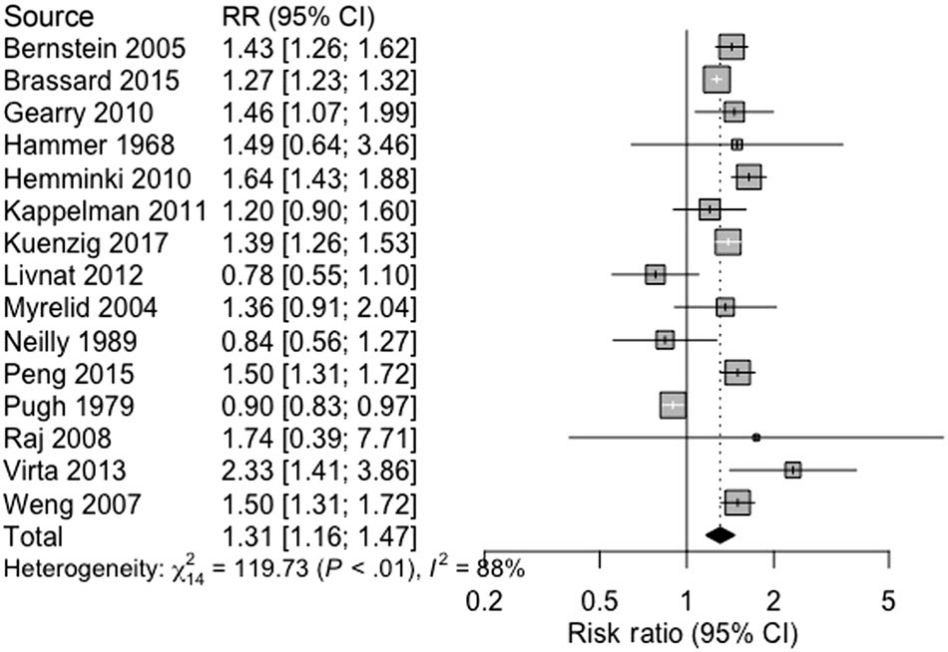
Association between asthma and Crohn’s disease .

**Fig. 3:**
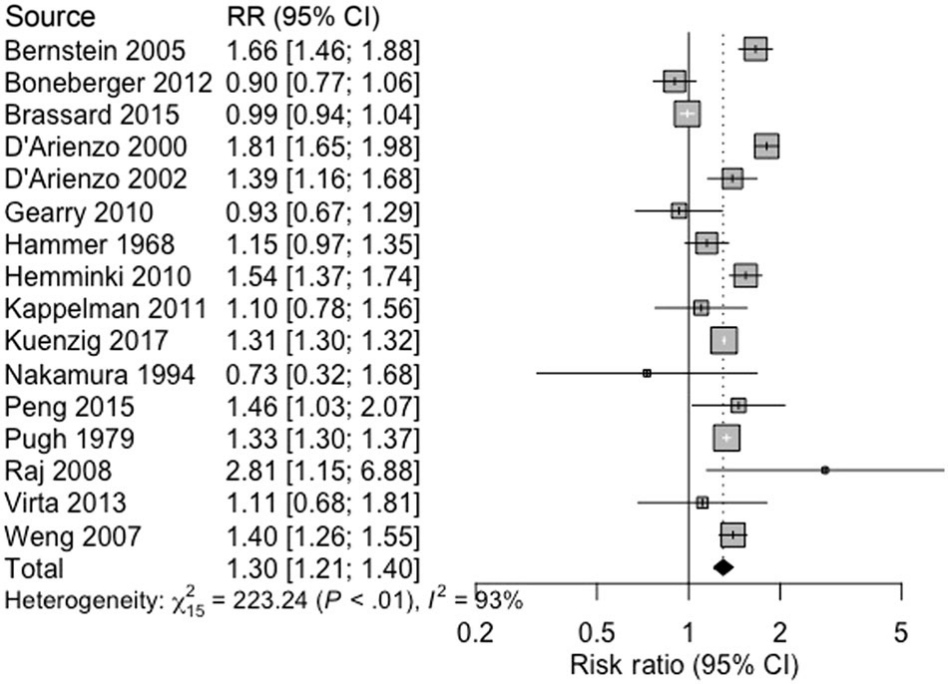
Association between asthma and ulcerative colitis .

Asthma and Crohn’s disease were associated (pooled RR 1.31, 95% CI 1.16 –1.47, 15 studies, 824,173 participants; heterogeneity: *I*^2^ = 88%, *p* < 0.0001; Fig. 2). There was a significant association between UC and asthma (pooled RR 1.30, 95% CI 1.21–1.40, 16 studies, 819,714 participants; heterogeneity: *I*^2^ = 93%, *p* < 0.0001; Fig. 3). Asthma and IBD-U were associated in a single study (OR 1.9, 95% CI 1.6–2.4, 51,459 participants)^27^.

### Explaining heterogeneity: study design

The association between asthma and IBD was consistently elevated in both case-control and cohort studies (Table 2). Although 22% of heterogeneity was accounted for by study design in studies evaluating the association between asthma and UC, significant heterogeneity persisted (residual heterogeneity: *I*^2^ = 91%, *p* < 0.0001). Separately analyzing case-control and cohort studies in Crohn’s disease did not account for any heterogeneity (residual heterogeneity: *I*^2^ = 88%, *p* < 0.0001).

**Table 2:** Results of subgroup analyses and meta-regression based on study design

Type of IBD	Study design	RR (95% CI)	Heterogeneity	Number of studies	Number of participants	Subgroup differences	Residual heterogeneity
Crohn’s disease	Case-control	1.25 (1.05–1.50)	*I*^2^ = 89%	11	513,008	*p* = 0.24 REF	*I*^2^ = 69% *R*^2^ = 0% *p* < 0.0001
	Cohort	1.45 (1.23–1.71)	*I*^2^ = 82%	4	311,165	*β* 1.6 (95% CI−0.14 to 0.46)	
Ulcerative colitis	Case-control	1.33 (1.24–1.42)	*I*^2^ = 89%	12	512,807	*p* = 0.78 REF	*I*^2^ = 91% *R*^2^ = 22% *p* < 0.0001
	Cohort	1.40 (0.99–1.98)	*I*^2^ = 94%	4	306,907	*β* −0.046 (95% CI −0.21 to 0.12)	

### Explaining heterogeneity: data source

Among studies evaluating the association between asthma and Crohn’s disease, there was a significantly elevated association in population-based studies (pooled RR 1.43, 95% CI 1.32–1.56, 10 studies, 822,775 participants; heterogeneity: *I*^2^ = 69%) but a protective association in studies recruiting patients from tertiary-care centers (pooled RR 0.89, 95% CI 0.83–0.97, 5 studies, 1398 participants; heterogeneity: *I*^2^ = 0%). Meta-regression based on source of patients (population-based or tertiary-care) accounted for 77% of heterogeneity among studies evaluating the association between asthma and Crohn’s disease. However, significant heterogeneity between studies remained (residual heterogeneity: *I*^2^ = 59%, *p* < 0.0001). No heterogeneity could be accounted for in studies evaluating the association between asthma and ulcerative colitis (residual heterogeneity: *I*^2^ = 94%, *p* < 0.0001). Results of meta-regression and subgroup analyses based on source of patients are summarized in Table 3.

**Table 3:** Results of subgroup analyses and meta-regression based on source of study participants

Type of IBD	Data source	RR (95% CI)	Heterogeneity	Number of studies	Number of participants	Subgroup differences	Residual heterogeneity
Crohn’s disease	Population-based or health maintenance organization	1.43 (1.32–1.56)	*I*^2^ = 69%	10	822,775	*p* < 0.0001 REF	*I*^2^ = 59% *R*^2^ = 77% *p* = 0.0024
	Tertiary-care center, not stated, or other	0.89 (0.83–0.97)	*I*^2^ = 0%	5	1398	*β* −0.48 (95% CI −0.65 to −0.30)	
Ulcerative colitis	Population-based or health maintenance organization	1.29 (1.13–1.46)	*I*^2^ = 94%	9	816,528	*p* = 0.94 REF	*I*^2^ = 94% *R*^2^ = 0% *p* < 0.0001
	Tertiary-care center, not stated, or other	1.30 (1.08–1.56)	*I*^2^ = 92%	7	3186	*β* 0.012 (95% CI −0.20 to 0.22)	

### Explaining heterogeneity: location of study

Differences across countries were observed in the association between asthma and Crohn’s disease (*p* < 0.0001). Between country differences accounted for 92% of the heterogeneity across case-control studies (residual heterogeneity: *I*^2^ = 33%, *p* = 0.16) evaluating the association between asthma and Crohn’s disease (Table 4).

**Table 4:** Results of subgroup analyses and meta-regression based on study location

Type of IBD	Country	RR (95% CI)	Heterogeneity	Number of studies	Number of participants	Subgroup differences	Residual heterogeneity
Crohn’s Disease	Canada	1.34 (1.24–1.45)	*I*^2^ = 64%	3	588,067	*p* < 0.0001 REF	*I*^2^ = 33% *R*^2^ = 92% *p* = 0.16
	Finland	2.33 (1.41–3.86)	–	1	1165	*β* 0.55 (0.036 to 1.07)	
	Israel	0.78 (0.55–1.10)	–	1	47	*β* −0.54 (−0.91 to −0.17)	
	New Zealand	1.46 (1.07–1.99)	–	1	1238	*β* 0.087 (−0.25 to 0.42)	
	Sweden	1.61 (1.41–1.83)	*I*^2^ = 0%	2	148,347	*β* 0.18 (0.0014 to 0.35)	
	Taiwan	1.50 (1.31–1.72)	–	1	25,799	*β* 0.11 (−0.071 to 0.30)	
	United Kingdom	0.90 (0.83–0.97)	*I*^2^ = 0%	4	1351	*β* −0.39 (−0.54 to −0.25)	
	United States	1.39 (1.13–1.71)	*I*^2^ = 47%	2	57,159	*β* 0.062 (−0.10 to 0.23)	
Ulcerative colitis	Canada	1.28 (1.03–1.60)	*I*^2^ = 98%	3	583,902	*p* < 0.0001 REF	*I*^2^ = 96% *R*^2^ = 0% *p* < 0.0001
	Chile	0.90 (0.77–1.06)	–	1	226	*β* −0.35 (−0.80 to 0.099)	
	Finland	1.11 (0.68–1.81)	–	1	1810	*β* −0.14 (−0.79 to 0.50)	
	Italy	1.61 (1.25–2.07)	*I*^2^ = 84%	2	182	*β* 0.22 (−0.13 to 0.57)	
	Japan	0.73 (0.32–1.68)	–	1	768	*β* −0.56 (−1.50 to 0.37)	
	New Zealand	0.93 (0.67–1.29)	–	1	1253	*β* −0.32 (−0.85 to 0.21)	
	Sweden	1.54 (1.37–1.74)	–	1	148,295	*β* 0.18 (−0.26 to 0.62)	
	Taiwan	1.46 (1.03–2.07)	–	1	21,541	*β* 0.13 (−0.42 to 0.68)	
	United Kingdom	1.29 (1.09–1.54)	*I*^2^ = 66%	3	2010	*β* 0.031 (−0.31 to 0.37)	
	United States	1.32 (1.07–1.62)	*I*^2^ = 41%	2	59,727	*β* 0.0018 (−0.37 to 0.37)	

Similarly, differences between countries were noted for the association between asthma and UC (*p* < 0.0001; Table 4). However, no heterogeneity could be accounted for by country (residual heterogeneity: *I*^2^ = 96%; *p* < 0.0001).

### Sensitivity analysis: relative timing of diagnoses

When restricting the analysis to studies in which the diagnosis of asthma preceded the diagnosis of Crohn’s disease, there was a significant association (pooled RR 1.49, 95% CI 1.27–1.74, 4 studies, 691,525 participants; heterogeneity: *I*^2^ = 86%, *p* < 0.0001; Supplementary Figure 1). In studies in which the diagnosis of asthma preceded the diagnosis of UC, the two diseases were not significantly associated (pooled RR 1.21, 95% CI 0.98–1.51, 5 studies, 692,228 participants; *I*^2^ = 97%, *p* < 0.0001; Supplementary Figure 2).

Patients previously diagnosed with both Crohn’s disease and UC were at an increased risk of new-onset asthma in a single study (Crohn’s disease: hazard ratio (HR) 1.50, 95% CI 1.31–1.72; UC: HR 1.46, 95% CI 1.03–2.07)^29^.

### Subgroup analysis: age at IBD diagnosis

The impact of age at IBD diagnosis on the association between asthma and IBD was evaluated in three studies: one case-control study limited to the association between asthma and UC (408,264 participants)^22^, and one cohort study evaluating the association between asthma and both Crohn’s disease and UC (136,178 participants)^42^, and one cohort study evaluating the association between asthma and IBD (26,300 participants)^29^. An additional three studies were limited to pediatric patients and were included in subgroup analyses of pediatric-onset IBD (7554 participants)^26,44,45^. There was no association between asthma and pediatric-onset Crohn’s disease (pooled RR 1.35, 95% CI 0.94–1.93; heterogeneity: *I*^2^ = 92%, Supplementary Figure 3). Adult-onset Crohn’s disease was associated with asthma (20–29 years at diagnosis: incident rate ratio (IRR) 1.18, 95% CI 1.10–1.26; 30–39 years: IRR 1.42, 95% CI 1.32–1.53; 40–49 years: IRR 1.31, 95% CI 1.23–1.41). Pooling across age groups in early adulthood (20–29 and 30–39 years), Crohn’s disease and asthma were associated in adults diagnosed in young adulthood (pooled RR 1.29, 95% CI 1.08–1.55; heterogeneity: *I*^2^ = 92%). Meta-regression based on age group did not account for any heterogeneity (residual heterogeneity: *I*^2^ = 92%, *p* < 0.0001).

Asthma and UC were associated in patients diagnosed with UC as young adults (≤40 years: pooled RR 1.11, 95% CI 1.04–1.19; *I*^2^ = 0%; Supplementary Figure 4). There was no association between asthma and UC diagnosed during childhood (pooled RR 1.11, 95% CI 0.97–1.28; *I*^2^ = 0%) or after 40 years of age (pooled RR 1.07, 95% CI 0.57–2.00; *I*^2^ = 98%). Among studies analyzing the association between asthma and UC in those diagnosed as older adults, one study suggested there was an increased association while the other identified a protective association. Significant heterogeneity persisted following meta-regression based on age groups (residual heterogeneity: *I*^2^ = 87%, *p* < 0.0001).

## Discussion

This systematic review and meta-analysis suggests the frequent co-occurrence of asthma with both Crohn’s disease and UC. IBD has also been associated with other respiratory disorders (e.g., chronic obstructive pulmonary disease)^42,54^. Further, both asthma and IBD have been associated with other immune-mediated and atopic conditions, including multiple sclerosis, rheumatoid arthritis, diabetes, eczema, and rhinitis^28,44,46,48,55–59^. However, there was a high degree of heterogeneity between studies, suggesting that the association between asthma and IBD may vary across populations and be impacted by differences in study methodology.

The association between Crohn’s disease and asthma was consistent regardless of the relative timing of the diagnoses of the two diseases (i.e., asthma preceding Crohn’s disease or Crohn’s disease preceding asthma). However, the risk of asthma was elevated among patients with existing UC but patients with existing asthma did not appear to be at an increased risk of UC. Based on these findings, it is not clear if one disease results in a predisposition to the other or the co-occurrence of these diseases simply occurs due to the commonalities in the physiology of the gut and the lung, as well as shared genetic and environmental risk factors.

Analyses stratified by age at diagnosis of IBD were inconsistent, with some studies suggesting a consistently elevated association between the two diseases across all ages, while others found contradictory age-specific associations. For example, one study found that the association between asthma and UC was decreased among people who were diagnosed with UC between 40 and 49 years of age^42^, while another study found an increased risk among patients diagnosed with UC > 40 years of age^22^. As both studies used health administrative data, and neither used an internally validated algorithm to identify cases of asthma, misclassification of asthma may have resulted in bias.

Additionally, failure to account for smoking status may have influenced the findings of these studies. Smoking is associated with an increased risk of Crohn’s disease but a decreased risk of UC^60^. Of the three included studies that adjusted for smoking status, two reported elevated associations between asthma and Crohn’s disease while one suggested that there may be a protective effect of the two diseases but was underpowered to detect a difference^24,27,47^. Asthma and UC were associated in one study that adjusted for smoking but not the other^24,27^. Differential rates of smoking across ages at IBD diagnosis may also contribute to differences observed in the association between asthma and IBD. For example, smoking is significantly more common among patients who are older at the time of Crohn’s disease diagnosis^61^. As a result, smokers with Crohn’s disease may be at an elevated risk of respiratory disease due to smoking but not their IBD. The decreased rate of smoking among patients with UC may explain the protective association seen in one study^42^. Similarly, other environmental risk factors (e.g., air pollution) demonstrate age-specific associations with both asthma and IBD and may contribute to the differences in the age-specific associations between asthma and IBD^62,63^.

A limitation of studies evaluating the associations between two conditions is that patients diagnosed with one condition have higher health services utilization than healthy individuals and may subsequently be more likely to be diagnosed with another condition^64^. No study included in our systematic review accounted for increased health care use. As a result, our findings of an increased association between asthma and IBD may have resulted from detection bias.

The association between asthma and Crohn’s disease varied across geographic regions. In Canada, New Zealand, Finland, Sweden, Taiwan, and the United States, the association between asthma and Crohn’s disease was elevated. However, there was a negative association in the United Kingdom and Israel. Regional differences in the association between asthma and UC were less pronounced. Although the reasons for these regional differences are not known, it is possible that differing penetrance of genetic or environmental risk factors may contribute. For example, the high prevalence of early-life exposure to peanuts has been associated with a decreased risk of peanut allergy in Israel^65^. This decreased risk of atopy may also result in a decreased risk of asthma among these children but not impact their elevated risk of developing IBD, due to genetic predisposition of IBD amongst Ashkenazi Jews.

As this is a systematic review, our ability to make conclusions about the co-occurrence of asthma and IBD is limited by the availability and quality of previous studies evaluating the association between these two diseases. Although we included 19 studies in our review, there was high degree of heterogeneity across studies. Heterogeneity was reduced when accounting for study methodology (i.e., study design and source of study participants) in studies analyzing the association between asthma and Crohn’s disease but not in the association between asthma and UC. In fact, there was an elevated association between asthma and Crohn’s disease in population-based studies, but a protective association between the two diseases in studies conducted using patients recruited from tertiary-care centers. All samples recruiting patients from tertiary-care centers were conducted either in Israel or the United Kingdom. This suggests that there are other underlying differences between studies and populations when it comes to understanding the association between asthma and IBD and we are unable to ascertain if the differences we observed result from differing study design or differences across populations. Specifically, the majority of studies recruiting patients from tertiary-care centers may have introduced selection bias by their choice of controls (e.g., partners of cases). However, similar control groups were selected in studies evaluating the association between UC and asthma included control groups that could similarly have introduced selection bias, yet the association remained similar regardless of the source of patients. In addition, there may be other differences in study design that were not accounted for (e.g., differences in the case definitions for IBD and asthma and variables adjusted for), underlying differences in the study population, or the phenotypes of asthma and IBD identified in the study (i.e., disease severity, behavior, management approach).

## Conclusions

Asthma is associated with both Crohn’s disease and ulcerative colitis. Geographic differences, as well as differences in study design, contribute to this heterogeneity. We were unable to determine whether one disease increases the risk of the other or if both arise due to commonalities in pathology and shared risk factors. Future well-designed observational research should attempt to address these issues in their study design.

## Study Highlights

### What is current knowledge

- Asthma and the inflammatory bowel diseases share environmental, genetic, and microbial risk factors

### What is new here

- Asthma is associated with both Crohn’s disease and ulcerative colitis
- The relationship between these two diseases appears to vary by region

## Electronic supplementary material

Supplementary Information
