# Supplementary material for: Co-occurrence of Asthma and the Inflammatory Bowel Diseases: A Systematic Review and Meta-analysis
Source: Clin Transl Gastroenterol. 2018 Sep 24;9(9):188. doi: 10.1038/s41424-018-0054-z (PMC6155154; doi:10.1038/s41424-018-0054-z)
Supplement: Supplementary file 1 — Supplementary Information [file 41424_2018_54_MOESM1_ESM.docx]

**Supplementary Table 1. Search strategy used to identify studies evaluating the association between asthma and the inflammatory bowel diseases.**

| **Ovid MEDLINE(R) Epub Ahead of Print, In-Process & Other Non-Indexed Citations, Ovid MEDLINE(R) Daily and Ovid MEDLINE(R)**1946 to Present |
| --- |
| 1. exp asthma/ 2. asthma$.mp. 3. (antiasthma$ or anti-asthma$).mp. 4. Respiratory sounds/ 5. wheez$.mp. 6. Bronchial Spasm/ 7. bronchospas$.mp. 8. (bronch$ adj3 spasm$).mp. 9. bronchoconstrict$.mp. 10. Bronchoconstriction/ 11. (bronch$ adj3 constrict$).mp. 12. Bronchial Hyperreactivity/ 13. Respiratory Hypersensitivity/ 14. ((bronchial$ or respiratory or airway$ or lung$) adj3 (hypersensitiv$ or hyperreactiv$ or allerg$ or insufficien$)).mp. 15. ((dust or mite$) adj3 (allerg$ or hypersensitiv$)).mp. 16. or/1-15 17. exp inflammatory bowel diseases/ 18. exp crohn disease/ 19. exp colitis, ulcerative/ 20. exp enterocolitis/ 21. exp proctitis/ 22. exp ileitis/ 23. crohn*.mp. 24. ulcerative colitis.mp. 25. "inflammatory bowel disease*".mp. 26. 17 or 18 or 19 or 20 or 21 or 22 or 23 or 24 or 25 27. 16 and 26 |
| EMBASE |
| 1. exp asthma/ 2. asthma$.mp. 3. (antiasthma$ or anti-asthma$).mp. 4. Abnormal Respiratory Sound/ 5. Wheezing/ 6. wheez$.mp. 7. bronchospasm/ 8. bronchospas$.mp. 9. (bronch$ adj3 spasm$).mp. 10. exp Bronchoconstriction/ 11. bronchoconstrict$.mp. 12. Respiratory Tract Allergy/ 13. (bronch$ adj3 constrict$).mp. 14. Bronchus Hyperreactivity/ 15. House Dust Allergy/ 16. ((bronchial$ or respiratory or airway$ or lung$) adj3 (hypersensitiv$ or hyperreactiv$ or allerg$ or insufficien$)).mp. 17. ((dust or mite$) adj3 (allerg$ or hypersensitiv$)).mp. 18. or/1-17 19. exp inflammatory bowel disease/ 20. exp crohn disease/ 21. exp ulcerative colitis/ 22. exp enterocolitis/ 23. exp proctitis/ 24. exp ileitis/ 25. crohn*.mp. 26. ulcerative colitis.mp. 27. "inflammatory bowel disease*".mp. 28. or/19-27 29. 18 and 28 30. limit 29 to conference abstract 31. 29 not 30 |

**Supplementary Table 2. Reasons for exclusion**

| **Study** | **Reason for Exclusion** |
| --- | --- |
| Andreoletti 2015(1) | No control group |
| Anonymous 1985(2) | Did not report the association between asthma and IBD |
| Balzola 2010(3) | Review article |
| Balzola 2011(4) | Review article |
| Balzola 2011(5) | Review article |
| Balzola 2013(6) | Review article |
| Bernstein 2008(7) | Meeting summary |
| Bhat 2007(8) | Case report |
| Ceyhan 2015(9) | Rate of asthma in the control group not reported |
| Chandra 2011(10) | Did not report the association between asthma and IBD |
| Chen 2017(11) | Did not report the association between asthma and IBD |
| Colldahl 1965(12) | Case report |
| Conway 2017(13) | No control group |
| D’Amato 2007(14) | Did not report the association between asthma and IBD |
| D’Andrea 2010(15) | Review article |
| Desai 2011(16) | Assessed the impact of IBD on pulmonary function, not asthma |
| Edwards 2004(17) | Did not report the association between IBD and asthma |
| Eliakim 1996(18) | Review article |
| Fenta 2010(19) | Did not separate Crohn’s disease and ulcerative colitis |
| Gibbs 1971(20) | Cell line, animal or translational research |
| Grover 2014(21) | Did not report the association between asthma and IBD |
| Grünberger 1984(22) | Did not report the association between asthma and IBD |
| Grünberger 1985(23) | Did not report the association between asthma and IBD |
| Gut 2011(24) | Case report |
| Haapamäki 2011(25) | Combined asthma with COPD |
| Heinzmann 2003(26) | Did not report the association between asthma and IBD |
| Hemminki 2010(27) | Evaluated familial asthma as a risk factor for IBD |
| Herrlinger 2002(28) | Assessed the impact of IBD on pulmonary function, not asthma |
| Hirota 2015(29) | Letter to the editor |
| Huang 2014(30) | Cell line, animal, or translational research |
| Hubbard 2004(31) | Review article |
| Hutchings 2008(32) | Did not report the association between IBD and asthma |
| Hyams 1988(33) | Did not report the association between IBD and asthma |
| Iannello 2003(34) | Did not report the association between IBD and asthma |
| Ikonomi 2016(35) | Did not report the association between IBD and asthma |
| Ilonidis 2004(36) | Assessed the impact of IBD on pulmonary function, not asthma |
| Kabesch 2003(37) | Did not report the association between IBD and asthma |
| Kanazawa 2005(38) | Cell line, animal or translational research |
| Kaplan 2011(39) | Review article |
| Kauppi 2015(40) | Did not separate Crohn’s disease and ulcerative colitis |
| Koek 2002(41) | Assessed the impact of IBD on pulmonary function, not asthma |
| Koning 2013(42) | Did not evaluate the association between asthma and IBD |
| Li 2012(43) | Did not evaluate the association between asthma and IBD |
| Li 2014(44) | No control group |
| Louis 1995(45) | Assessed the impact of IBD on pulmonary function, not asthma |
| Louis 1999(46) | Assessed the impact of IBD on pulmonary function, not asthma |
| Majewski 2015(47) | Review article |
| Manguso 2002(48) | Duplicate of included study |
| Mansi 2000(49) | Assessed the impact of IBD on pulmonary function, not asthma |
| Mikhailova 2011(50) | Assessed the impact of IBD on pulmonary function, not asthma |
| Moes-Wójtowicz 2012(51) | No control group |
| Olén 2014(52) | Excluded patients with IBD |
| Parambil 2009(53) | Did not report the association between IBD and asthma |
| Park 2005(54) | Did not report the association between IBD and asthma |
| Pearson 1983(55) | Case report |
| Peradzynska 2012(56) | Rate of asthma in the control group not reported |
| Persson 1996(57) | Reports on asthma-specific mortality rates in patients with IBD |
| Poon 2004(58) | Cell line, animal or translational research |
| Ramagopalan 2013(59) | Did not report the association between IBD and asthma |
| Rodriguez-Roisin 2016(60) | Review article |
| Romano 2016(61) | Review article |
| Sarioglu 2006(62) | Letter to the editor |
| Sclano 2002(63) | Review article |
| Sharifpour 2012(64) | Excluded patients with pre-existing respiratory disease |
| Sibtain 2011(65) | Evaluated familial asthma as a risk factor for IBD |
| Sivagnanam 2007(66) | No control group |
| Sivagnanasundaram 2004(67) | Did not report the association between IBD and asthma |
| Skyring 1965(68) | No control group |
| Swynghedauw 2007(69) | Review article |
| Taherzadeh 2012(70) | Assessed the impact of IBD on pulmonary function, not asthma |
| Tanday 2015(71) | Review article |
| Tirosh 2006(72) | Did not separate Crohn’s disease and ulcerative colitis |
| Upton 2015(73) | Did not report the association between IBD and asthma |
| Van Limbergen 2009(74) | No control group |
| Vutcovici 2016(75) | Reported on mortality in co-occurring asthma and COPD |
| Wang 2013(76) | Review article |
| Weidinger 2005(77) | Evaluated the association between genetic factors and asthma |
| Yang 2000(78) | Did not report the association between IBD and asthma |
| Yun 2012(79) | Duplicate cohort of Fenta 2010(19) |

**Supplementary Table 3. Risk of bias of included case-control and cross-sectional studies**

| Study | Adequate Case Definition | Representativeness of the Cases | Selection of Controls | Controls Did Not Have Disease | Comparability of Cases and Controls | Ascertainment of Exposure | Same method of ascertainment for cases and controls | Non-response rate |
| --- | --- | --- | --- | --- | --- | --- | --- | --- |
| Bernstein 2005(80) | Yes, with independent validation | Consecutive or obviously representative series of cases (population-based) | Community controls | Yes | Study controls (adjusts) for important confounding variables | Secure record | Yes | Not relevant (administrative data) |
| Boneberger 2012(81) | No description | Consecutive or obviously representative series of cases (tertiary-care centre) | Hospital controls | Not reported | Study controls (adjusts) for important confounding variables | No description | Not reported | Response rates not reported |
| D'Arienzo 2000(82) | Yes, with independent validation | Consecutive or obviously representative series of cases (tertiary-care centre) | Other – Hospital staff | Yes | No control (adjustment) for potential confounding variables | Structured interview where blinded to case/control status | Yes | Response rates not reported |
| D’Arienzo 2002(83) | Yes, with independent validation | Consecutive or obviously representative series of cases (tertiary-care centre) | Other – Partners of cases | Yes | No control (adjustment) for potential confounding variables | Structured interview where blinded to case/control status | Yes | Response rates not reported |
| Gearry 2010(84) | Yes, with independent validation | Consecutive or obviously representative series of cases (population-based) | Community controls | Not reported | Study controls (adjusts) for important confounding variables | Written self-report or medical record only | Yes | Non respondents described |
| Hammer 1968(85) | Yes, with independent validation | Potential for selection biases or not stated (tertiary-care centre) | Hospital/clinic controls | Not reported | No control (adjustment) for potential confounding variables | Interview; unclear if blinded to case/control status | Yes | Not reported |
| Kappelman 2011(86) | Yes, with independent validation | Potential for selection biases or not stated (health maintenance organization) | Other – patients in same health maintenance organization without IBD | Yes | Study controls (adjusts) for important confounding variables | Secure record | Yes | Not relevant (administrative data) |
| Kuenzig 2017(87) | Yes, with independent validation | Consecutive or obviously representative series of cases (population-based) | Community Controls | Yes | Study controls (adjusts) for important confounding variables | Secure record | Yes | Not relevant (administrative data) |
| Livnat 2012(88) | Yes, with independent validation | Consecutive or obviously representative series of cases (tertiary-care centre) | Hospital/clinic controls | Not reported | No control (adjustment) for potential confounding variables | Written self report or medical record only | Yes | Cases: Non respondents described Controls: Response rates not reported |
| Myrelid 2004(89) | Yes, with independent validation | Potential for selection biases or not stated | Community controls | Not reported | Study controls (adjusts) for important confounding variables | Written self-report or medical record only | Yes | Same rate for both groups |
| Nakamura 1994(90) | Yes, with independent validation | Consecutive or obviously representative series of cases (population-based) | Hospital/clinic controls | Not reported | Study controls (adjusts) for important confounding variables | Written self report or medical record only | Yes | Response rates not reported |
| Neilly 1989(91) | No description | Potential for selection biases or not stated | No description | Not reported | Study controls (adjusts) for important confounding variables | Interview; unclear if blinded to case/control status | Yes | Same rate for both groups |
| Pugh 1979(92) | Yes, with independent validation | Potential for selection biases or not stated (tertiary-care centre; Ileostomy Association) | Other – Partners of cases (51%); hospital/clinic controls (49%) | Not reported | No control (adjustment) for potential confounding variables | Written self report or medical record only | Yes | Response rates not reported |
| Virta 2013(93) | Yes, with independent validation | Consecutive or obviously representative series of cases (population-based) | Community controls | Not reported | Study controls (adjusts) for potential confounding variables | Secure record | Yes | Response rates not relevant (e.g., administrative data) |
| Weng 2007(94) | Yes, with independent validation | Potential for selection biases or not stated (health maintenance organization) | Other – patients in same health maintenance organization without IBD | Yes | Study controls (adjusts) for important confounding variables | Secure records | Yes | Response rates not relevant (eg administrative data) |

**Supplementary Table 4. Risk of bias in cohort studies**

| Study | Representativeness of the Exposed Cohort | Selection of the Non-Exposed Cohort | Ascertainment of Exposure | Demonstration that outcome of interest was not present at start of study | Comparability of cohorts on the basis of the design or analysis | Assessment of Outcome | Sufficient follow-up | Adequacy of follow-up cohorts |
| --- | --- | --- | --- | --- | --- | --- | --- | --- |
| Brassard 2015(95) | Somewhat representative of the average rate of disease in the community^a^ | Drawn from the same community as the exposed cohort^b^ | Secure record | Yes | Study controls (adjusts) for important confounding variables | Record linkage | Yes | Not relevant (administrative data) |
| Hemminki 2010(96) | Somewhat representative of the average rate of disease (IBD or asthma) in the community | Drawn from the same community as the exposed cohort | Secure record | Yes | Study controls (adjusts) for important confounding variables | Record linkage | Yes | Not relevant (administrative data) |
| Peng 2015(97) | Truly representative of the average rate of disease (IBD or asthma) in the community | Drawn from the same community as the exposed cohort | Secure record | Yes | Study controls (adjusts) for important confounding variables | Record linkage | Yes | Not relevant (administrative data) |
| Raj 2008(98) | Selected group of users^c^ | Drawn from the same community as the exposed cohort^b^ | Secure record | No | No control (adjustment) for potential confounding variables | Record linkage | Yes | No statement |

^a^Study was conducted using provincial health administrative data, but only included patients with provincial pharmacare (age ≥65, on social assistance, or with no employee benefits)

^b^The rate of inflammatory bowel disease (IBD) among people with asthma was compared to the rate of asthma in the general population

^c^Patients with asthma were recruited from a tertiary-care respiratory centre

**Supplementary Figure 1. Sensitivity analysis restricted to studies in which the diagnosis of asthma preceded the diagnosis of Crohn’s disease**

**
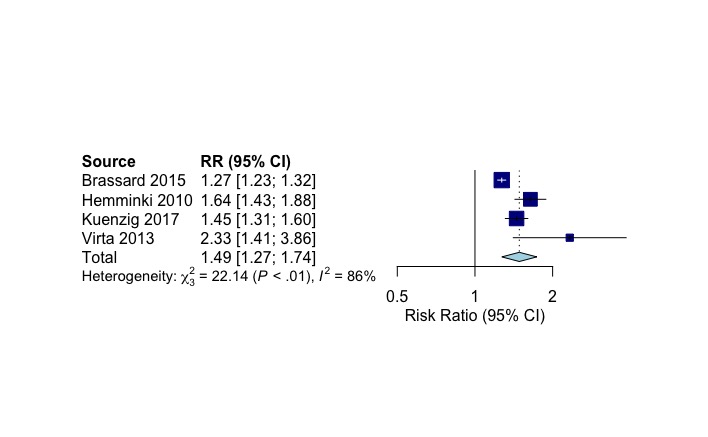
**

**Supplementary Figure 2. Sensitivity analysis restricted to studies in which the diagnosis of asthma preceded the diagnosis of ulcerative colitis**


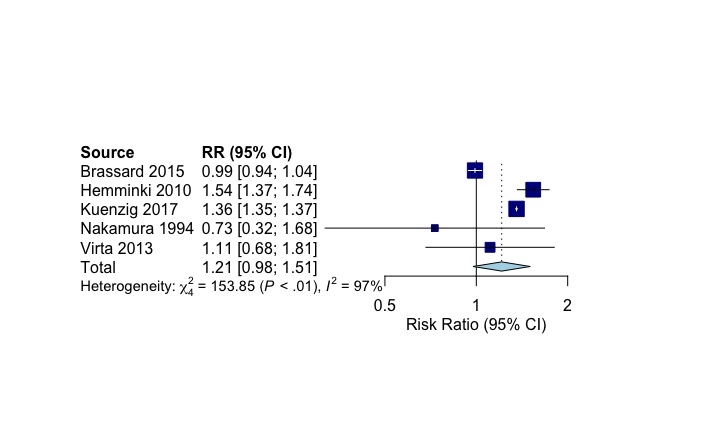


**Supplementary Figure 3. Association between asthma and Crohn’s disease stratified by age at IBD diagnosis**

**
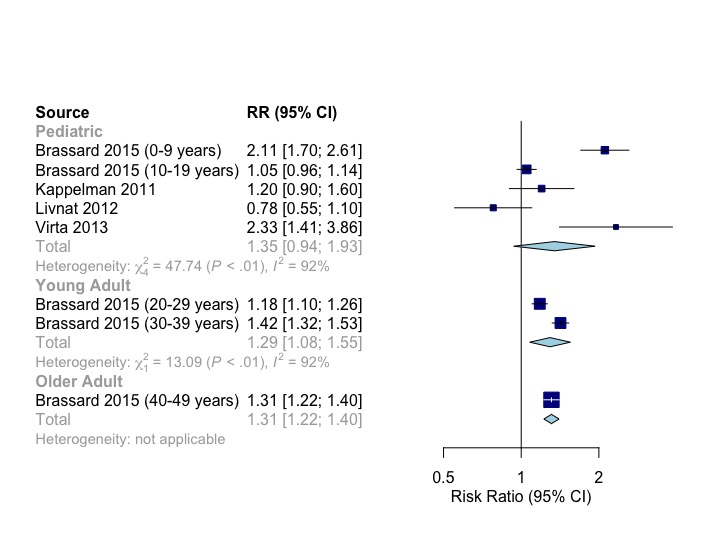
**

**Supplementary Figure 4. Association between asthma and ulcerative colitis stratified by age at IBD diagnosis**

**
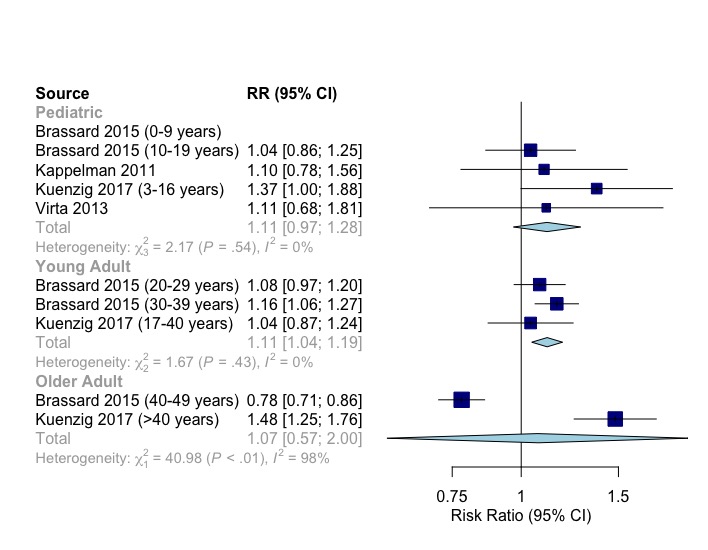
**

**References**

1. Andreoletti G, Ashton JJ, Coelho T, *et al.* Exome analysis of patients with concurrent pediatric inflammatory bowel disease and autoimmune disease. Inflamm Bowel Dis 2015;21:1229–1236.

2. Anonymous. Families with psychosomatically ill children. Study of family dynamics in bronchial asthma and ulcerative colitis [Familien mit psychosomatisch kranket Kindern. Familiedynamische untersuchungen zum Asthma bronchiale und zur Colitis ulcerosa]. 1985.

3. Balzola F, Bernstein C, Van Assche G. Familial association of inflammatory bowel diseases with other autoimmune and related diseases: Commentary. Inflamm Bowel Dis Monit 2010;10:91–92.

4. Balzola F, Bernstein C, Ho GT, *et al.* Pulmonary involvement in inflammatory bowel disease. Inflamm Bowel Dis Monit 2011;11:128–129.

5. Balzola F, Bernstein C, Ho GT. Increased risk for coronary heart disease, asthma, and connective tissue diseases in inflammatory bowel disease. Inflamm Bowel Dis Monit 2011;11:162.

6. Balzola F, Cullen G, Hoentjen F, *et al.* Comorbidity of allergic and autoimmune disease among patients with ADHD: A nationwide population-based study. Inflamm Bowel Dis Monit 2013;13:169–170.

7. Bernstein CN. Assessing environmental risk factors affecting the inflammatory bowel diseases: A joint workshop of the Crohnʼs & Colitis Foundations of Canada and the USA. Inflamm Bowel Dis 2008;14:1139–1146.

8. Bhat M, Dawson D. Wheezes, blisters, bumps and runs: multisystem manifestations of a Crohn's disease flare-up. CMAJ 2007;177:715–718.

9. Ceyhan BB, Karakurt S, Cevik H, *et al.* Bronchial Hyperreactivity and Allergic Status in Inflammatory Bowel Disease. Respiration 2003;70:60–66.

10. Chandra RK, Lin D, Tan B, *et al.* Chronic rhinosinusitis in the setting of other chronic inflammatory diseases. Am J Otolaryngol 2011;32:388–391.

11. Chen M-H, Su T-P, Chen Y-S, *et al.* Comorbidity of Allergic and Autoimmune Diseases Among Patients With ADHD. J Atten Disord 2017;21:219–227.

12. Colldahl H. Allergy and certain diseases in relation to the digestive tract: some observations on the effect of elimination diets. Acta Allergol 20:84–93.

13. Conway G, Velonias G, Andrews E, *et al.* The impact of co-existing immune-mediated diseases on phenotype and outcomes in inflammatory bowel diseases. Aliment Pharmacol Ther 2017;45:814–823.

14. D’Amato M, Bruce S, Bresso F, *et al.* Neuropeptide S Receptor 1 Gene Polymorphism Is Associated With Susceptibility to Inflammatory Bowel Disease. Gastroenterology 2007;133:808–817.

15. D'Andrea N, Vigliarolo R, Sanguinetti CM. Respiratory involvement in inflammatory bowel diseases. Multidiscip Respir Med 2010;5:173–182.

16. Desai D, Patil S, Udwadia Z, *et al.* Pulmonary manifestations in inflammatory bowel disease: a prospective study. Indian J Gastroenterol 2011;30:225–228.

17. Edwards LJ, Constantinescu CS. A prospective study of conditions associated with multiple sclerosis in a cohort of 658 consecutive outpatients attending a multiple sclerosis clinic. Mult Scler 2004;10:575–581.

18. Eliakim R, Rachmilewitz D. Inflammatory bowel disease: The asthma of the intestine? Inflamm Bowel Dis 1996;2:122–132.

19. Fenta YA, Tello N, Jung JA, *et al.* Inflammatory bowel disease and asthma: A population-based, case-control study [Internet]. Inflamm Bowel Dis 2010;16:1957–1962.Available from: https://academic.oup.com/ibdjournal/article/16/11/1957-1962/4628292

20. Gibbs NM, French LW. The colon mucus antibody. J. Clin. Pathol. 1971;24:867–869.

21. Grover Z, Biron R, Carman N, *et al.* Predictors of response to Infliximab in children with luminal Crohn's disease. J Crohns Colitis 2014;8:739–746.

22. Grünberger J, Linzmayer L, Gathmann P, *et al.* [Duration of spiral after-effects in psychosomatic patients: a psychophysiologic study and analysis of data using multivariate procedures]. Wien Klin Wochenschr 1984;96:592–597.

23. Grünberger J, Linzmayer L, Gathmann P, *et al.* [Computer-assisted static and light-evoked dynamic pupillometry in psychosomatic patients]. Wien Klin Wochenschr 1985;97:775–781.

24. Gut G, Sivan Y. Respiratory Involvement in Children with Inflammatory Bowel Disease. Pediatr Allergy Immunol Pulmonol 2011;24:197–206.

25. Haapamäki J, Roine RP, Turunen U, *et al.* Increased risk for coronary heart disease, asthma, and connective tissue diseases in inflammatory bowel disease. J Crohns Colitis 2011;5:41–47.

26. Heinzmann A. Association study of the IL13 variant Arg110Gln in atopic diseases and juvenile idiopathic arthritis. J Allergy Clin Immunol 2003;112:735–739.

27. Hemminki K, Li X, Sundquist K, *et al.* Familial association of inflammatory bowel diseases with other autoimmune and related diseases. Am J Gastroenterol 2010;105:139–147.

28. Herrlinger KR, Noftz MK, Dalhoff K, *et al.* Alterations in pulmonary function in inflammatory bowel disease are frequent and persist during remission. Am J Gastroenterol 2002;97:377–381.

29. Hirota JA, Carlsten C, Sadatsafavi M, *et al.* Airway diseases and inflammatory bowel diseases: is it something in the air (pollution)? Eur Respir J 2015;46:287–288.

30. Huang Y, Lemberg DA, Day AS, *et al.* Markers of Inflammation in the Breath in Paediatric Inflammatory Bowel Disease. J Pediatr Gastroenterol Nutr 2014;59:505–510.

31. Hubbard GP, Binion DG, Kelly KJ. What is the link between allergies and Crohn disease? J Respir Dis 2004;25:461–462.

32. Hutchings HA, Upton P, Cheung W-Y, *et al.* Development of a parent version of the Manchester-Minneapolis quality of life survey for use by parents and carers of UK children: MMQL-UK (PF). Health Qual Life Outcomes 2008;6:19–8.

33. Hyams JS, Moore RE, Leichtner AM, *et al.* Relationship of type I procollagen to corticosteroid therapy in children with inflammatory bowel disease. J Pediatr 1988;112:893–898.

34. Iannello S, Cavaleri A, Milazzo P, *et al.* Low fasting serum triglyceride level as a precocious marker of autoimmune disorders. MedGenMed 2003;5:20.

35. Ikonomi E, Rothstein RD, Ehrlich AC, *et al.* Measurement of fractional exhaled nitric oxide as a marker of disease activity in inflammatory bowel disease. J Gastroenterol Pancreatol Liv Disord 2016;3

36. Ilonidis G, Anogianakis G, Agorastos J, *et al.* Bronchial hyperresponsiveness in patients with inflammatory bowel disease. Eur J Inflamm 2004;2:119–123.

37. Kabesch M, Peters W, Carr D, *et al.* Association between polymorphisms in caspase recruitment domain containing protein 15 and allergy in two German populations. J Allergy Clin Immunol 2003;111:813–817.

38. Kanazawa H, Yoshikawa J. A case-control study of bronchial asthma associated with ulcerative colitis: role of airway microvascular permeability. Clin Exp Allergy 2005;35:1432–1436.

39. Kaplan G. Air pollution and the inflammatory bowel diseases. Inflamm Bowel Dis 2011;17:1146–1148.

40. Kauppi P, Linna M, Jantunen J, *et al.* Chronic Comorbidities Contribute to the Burden and Costs of Persistent Asthma. Mediators Inflamm 2015;2015:819194.

41. Koek GH, Verleden GM, Evenepoel P, *et al.* Activity related increase of exhaled nitric oxide in Crohn's disease and ulcerative colitis: a manifestation of systemic involvement? Respir Med 2002;96:530–535.

42. Koning M, Ailabouni R, Gearry RB, *et al.* Use and Predictors of Oral Complementary and Alternative Medicine by Patients With Inflammatory Bowel Disease. Inflamm Bowel Dis 2013;19:767–778.

43. Li X, Ampleford EJ, Howard TD, *et al.* Genome-wide association studies of asthma indicate opposite immunopathogenesis direction from autoimmune diseases. J Allergy Clin Immunol 2012;130:861–8.e7.

44. Li D, Collins B, Velayos FS, *et al.* Racial and Ethnic Differences in Health Care Utilization and Outcomes Among Ulcerative Colitis Patients in an Integrated Health-Care Organization. Dig Dis Sci 2014;59:287–294.

45. Louis E, Louis R, Drion V, *et al.* Increased frequency of bronchial hyperresponsiveness in patients with inflammatory bowel disease. Allergy 1995;50:729–733.

46. Louis R, Louis E, Shute J, *et al.* Bronchial eosinophilic infiltration in Crohn's disease in the absence of pulmonary disease. Clin Exp Allergy 1999;29:660–666.

47. Majewski S, Piotrowski W. Pulmonary manifestations of inflammatory bowel disease. Arch Med Sci 2015;6:1179–1188.

48. Manguso F, D'Arienzo A, Astarita C, *et al.* [Seronegative spondyloarthropathies and allergic diseases in patients with ulcerative colitis]. Reumatismo 2002;54:27–35.

49. Mansi A, Cucchiara S, Greco L, *et al.* Bronchial Hyperresponsiveness in Children and Adolescents with Crohn's Disease. Am J Respir Crit Care Med 2000;161:1051–1054.

50. Mikhailova ZF, Parfenov AI, Ruchkina IN, *et al.* External respiratory function in patients with Crohn's disease. Eksp Klin Gastroenterol :82–85.

51. Moes-Wójtowicz A, Wójtowicz P, Postek M, *et al.* Asthma as a psychosomatic disorder: the causes, scale of the problem, and the association with alexithymia and disease control. Pneumonol Alergol Pol 2012;80:13–19.

52. Olén O, Neuman Å, Koopmann B, *et al.* Allergy-related diseases and recurrent abdominal pain during childhood - a birth cohort study. Aliment Pharmacol Ther 2014;40:1349–1358.

53. Parambil JG, Yi ES, Ryu JH. Obstructive bronchiolar disease identified by CT in the non-transplant population: Analysis of 29 consecutive cases. Respirology 2009;14:443–448.

54. Park YR, Choi SC, Lee ST, *et al.* The association of eotaxin-2 and eotaxin-3 gene polymorphisms in a Korean population with ulcerative colitis. Exp Mol Med 2005;37:553–558.

55. Pearson DJ, Stones NA, Bentley SJ. Proctocolitis induced by salicylate and associated with asthma and recurrent nasal polyps. Br Med J (Clin Res Ed) 1983;287:1675–1675.

56. Peradzyńska J, Krenke K, Lange J, *et al.* Low prevalence of pulmonary involvement in children with inflammatory bowel disease. Respir Med 2012;106:1048–1054.

57. Persson P-G, Bernell O, Leijonmarck CE, *et al.* Survival and cause-specific mortality in inflammatory bowel disease: a population-based cohort study. Gastroenterology 1996;110:1339–1345.

58. Poon AH, Laprise C, Lemire M, *et al.* Association of vitamin D receptor genetic variants with susceptibility to asthma and atopy. Am J Respir Crit Care Med 2004;170:967–973.

59. Ramagopalan SV, Goldacre R, Disanto G, *et al.* Hospital admissions for vitamin D related conditions and subsequent immune-mediated disease: record-linkage studies. BMC Med 2013;11:171.

60. Rodriguez-Roisin R, Bartolome SD, Huchon G, *et al.* Inflammatory bowel diseases, chronic liver diseases and the lung. Eur Respir J 2016;47:638–650.

61. Romano C, Cardile S. Pulmonary implications in inflammatory bowel disease: not a rare event. Expert Opin Drug Saf 2016;15:1001–1002.

62. Sarioğlu N, Türkel N, Sakar A, *et al.* Lung involvement in inflammatory bowel diseases. Ann Saudi Med 2006;26:407–408.

63. Sclano G. Asthma, nasal polyposis and ulcerative colitis: a new perspective. Clin Exp Allergy 2002;32:1144–1149.

64. Sharifpour A, Fakheri HT, Aliali M, *et al.* Evaluation of correlation between ulcerative colitis with asthma and air way hyper responsiveness. J Mazand Univ Med Sci 2010;20:21–28.

65. Sibtain AM, Spady D, El-Matary W. Immune-related disorders in families of children with inflammatory bowel disease - A prospective cohort study. Ital J Pediatr 2011;37:49.

66. Sivagnanam P, Koutsoumpas A, Forbes A. Respiratory symptoms in patients with inflammatory bowel disease and the impact of dietary salicylates. Dig Liver Dis 2007;39:232–239.

67. Sivagnanasundaram S, Broman KW, Liu M, *et al.* Quasi-linkage: a confounding factor in linkage analysis of complex diseases? Hum Genet 2004;114:588–593.

68. Skyring A, Roberts R. Childhood ulcerative colitis: an epidemiological study in NSW. Med J Aust 1965;1:955–960.

69. Swynghedauw B. L’inflammation? Au cœur du problème médical. Arch Mal Coeur Vaiss Pratique 2007;2007:27–28.

70. Taherzadeh M, Vahedi H, Moghadam KG, *et al.* Pulmonary function and methacholine challenge tests in patients with ulcerative colitis. Tanaffos 2012;11:23–27.

71. Tanday S. Patients waking up to the benefits of interrupted sedation Patients with lung disease at risk of bowel disease. Lancet Respir Med 2014;3:16.

72. Tirosh A, Mandel D, Mimouni FB, *et al.* Autoimmune Diseases in Asthma. Ann Intern Med 2006;144:877–8.

73. Upton P, Eiser C, Cheung I, *et al.* Health and Quality of Life Outcomes. Health Qual Life Outcomes 2005;3:22–7.

74. Van Limbergen J, Russell RK, Nimmo ER, *et al.* Filaggrin loss-of-function variants are associated with atopic comorbidity in pediatric inflammatory bowel disease. Inflamm Bowel Dis 2009;15:1492–1498.

75. Vutcovici M, Bitton A, Ernst P, *et al.* Inflammatory bowel disease and risk of mortality in COPD. Eur Respir J 2016;47:1357–1364.

76. Wang H. Gut-lung crosstalk in pulmonary involvement with inflammatory bowel diseases. World J Gastroenterol 2013;19:6794–12.

77. Weidinger S, Klopp N, Rummler L, *et al.* Association of CARD15 polymorphisms with atopy-related traits in a population-based cohort of Caucasian adults. Clin Exp Allergy 2005;35:866–872.

78. Yang P, Tremaine WJ, Meyer RL, *et al.* α1-Antitrypsin Deficiency and Inflammatory Bowel Diseases. Mayo Clin Proc 2000;75:450–455.

79. Yun HD, Knoebel E, Fenta Y, *et al.* Asthma and Proinflammatory Conditions: A Population-Based Retrospective Matched Cohort Study. Mayo Clin Proc 2012;87:953–960.

80. Bernstein CN, Wajda A, Blanchard JF. The clustering of other chronic inflammatory diseases in inflammatory bowel disease: a population-based study. Gastroenterology 2005;129:827–836.

81. Boneberger A, Weiss EH, Calvo M, *et al.* Atopic manifestations in patients with ulcerative colitis: a report from Chile. J Investig Allergol Clin Immunol 2012;22:73–75.

82. D'Arienzo A. Allergy and Mucosal Eosinophil Infiltrate in Ulcerative Colitis. Scand J Gastroenterol 2000;35:624–631.

83. D'Arienzo A, Manguso F, Scarpa R, *et al.* Ulcerative Colitis, Seronegative Spondyloarthropathies and Allergic Diseases: the Search for a Link. Scand J Gastroenterol 2002;37:1156–1163.

84. Gearry RB, Richardson AK, Frampton CM, *et al.* Population-based cases control study of inflammatory bowel disease risk factors. J Gastroenterol Hepatol 2010;25:325–333.

85. Hammer B, Ashurst P, Naish J. Diseases associated with ulcerative colitis and Crohn's disease. Gut 1968;9:17–21.

86. Kappelman MD, Galanko JA, Porter CQ, *et al.* Association of paediatric inflammatory bowel disease with other immune-mediated diseases. Arch Dis Child 2011;96:1042–1046.

87. Kuenzig ME, Barnabe C, Seow CH, *et al.* Asthma is Associated With Subsequent Development of Inflammatory Bowel Disease: a Population-based Case–Control Study. Clin Gastroenterol Hepatol 2017;

88. Livnat G, Lerner A, Hakim F, *et al.* Bronchial reactivity and fractional exhaled NO in Crohn’s disease in the era of immunomodulating treatment. Acta Paediatr 2012;101:e399–e404.

89. Myrelid P, Dufmats M, Lilja I, *et al.* Atopic manifestations are more common in patients with Crohn disease than in the general population. Scand J Gastroenterol 2009;39:731–736.

90. Nakamura Y, Kobayashi M, Nagai M, *et al.* A case-control study of ulcerative colitis in Japan. J Clin Gastroenterol 1994;18:72–79.

91. Neilly JB, Main AN, McSharry C, *et al.* Pulmonary abnormalities in Crohn's disease. Respir Med 1989;83:487–491.

92. Pugh SM, Rhodes J, Mayberry JF, *et al.* Atopic disease in ulcerative colitis and Crohn's disease. Clin Allergy 1979;9:221–223.

93. Virta LJ, Ashorn M, Kolho K-L. Cowʼs Milk Allergy, Asthma, and Pediatric IBD. J Pediatr Gastroenterol Nutr 2013;56:649–651.

94. Weng X, Liu L, Barcellos LF, *et al.* Clustering of Inflammatory Bowel Disease With Immune Mediated Diseases Among Members of a Northern California-Managed Care Organization. Am J Gastroenterol 2007;102:1429–1435.

95. Brassard P, Vutcovici M, Ernst P, *et al.* Increased incidence of inflammatory bowel disease in Québec residents with airway diseases. Eur Respir J 2015;45:962–968.

96. Hemminki K, Li X, Sundquist J, *et al.* Subsequent Autoimmune or Related Disease in Asthma Patients: Clustering of Diseases or Medical Care? Ann Epidemiol 2010;20:217–222.

97. Peng Y-H, Liao W-C, Su C-H, *et al.* Association of inflammatory bowel disease with asthma risk: A nationwide cohort study. Allergy Asthma Proc 2015;36:92–98.

98. Raj AA, Birring SS, Green R, *et al.* Prevalence of inflammatory bowel disease in patients with airways disease. Respir Med 2008;102:780–785.
